# An Exploration of Smoking Patterns Among People with Serious Mental Illness Attending an Outpatient Clinic in Qatar

**DOI:** 10.2147/NDT.S385970

**Published:** 2022-12-07

**Authors:** Monica Zolezzi, Safa Al-Rawi, Yassin Eltorki

**Affiliations:** 1 Clinical Pharmacy and Practice, College of Pharmacy, QU Health, Qatar University, Doha, Qatar; 2 Al Wakrah Hospital, Hamad Medical Corporation (HMC), Doha, Qatar; 3 Mental Health Hospital, HMC, Doha, Qatar

**Keywords:** smoking, smoking cessation, serious mental illness, Qatar

## Abstract

**Background:**

Studies have shown that tobacco use is exceptionally high in people affected with serious mental illness (SMI). Many countries worldwide have observed a decrease in the prevalence of tobacco smoking; however, the smoking rates among people with SMI have declined much less than in those without mental illness. To date, no nationally representative data have examined the smoking patterns or the sociocultural factors that influence smoking among SMI people in Qatar.

**Methods:**

A retrospective medical chart review was conducted to collect patient demographics, psychiatric and medical comorbidities, medications, the most recently documented smoking status and if on tobacco cessation treatment. A descriptive and inferential analysis of the data was performed.

**Results:**

Of 346 patients included in the cohort, 196 (56.6%) had their smoking status documented, of which 72 (36.7%) were “currently smoking.” Significantly more males than females were “current smokers” (62.9% versus 15.0%, respectively, p < 0.001). Significantly more patients with psychotic disorders than those with any other SMI were “current smokers”, and this difference was statistically significant (p = 0.006). Positive and significant associations with current smoking were found for the male gender, psychotic disorders, and high levels (≥6.2 mmol/L) of total cholesterol. Only 12 (16.7%) of current smokers were receiving smoking cessation treatment.

**Conclusion:**

More than half of a sample of people with SMI attending outpatient psychiatric services in Qatar had documented smoking status. Still, only a few current smokers were on smoking cessation treatment. Efforts are needed to implement smoking cessation strategies in this population.

## Introduction

A substantial mortality gap exists in people with serious mental illness (SMI). Several systematic reviews have indicated that people with SMI die 13–30 years younger than their counterparts in the general population.^1^,^2^ A high proportion of this excess mortality is attributable to physical illnesses and modifiable risk factors, such as higher smoking rates relative to the general population.^3–6^ Studies have shown that tobacco use is exceptionally high in schizophrenia, with a prevalence rate of 62% reported in a meta-analysis.^7^ Studies have also seen high smoking rates in bipolar disorder, major depression disorder, and substance use disorders.^8–10^

The higher smoking prevalence in this population has been hypothesized to be due to various factors, such as genetic predisposition, having poor coping strategies to deal with psychiatric symptoms, and being socially reinforced in mental health facilities.^10^ Moreover, smokers with psychiatric illnesses tend to be heavier smokers, are considered more nicotine-dependent, and have been reported to suffer from more significant withdrawal symptoms upon smoking cessation.^10^,^11^ There is also evidence that nicotine interferes with antipsychotic medications by increasing the metabolism of some psychiatric drugs such as antipsychotics, potentially decreasing their blood levels and therapeutic effect.^12^,^13^ Studies have also reported that compared to non-smokers, adult tobacco users typically have unhealthy eating patterns, poorer diets and lower body mass indices (BMIs).^14^,^15^ These studies have also shown that smoking cessation is linked to an overall weight gain.

Many countries worldwide have observed a decrease in the prevalence of tobacco smoking; however, the smoking rates among people with SMI have declined much less than in those without mental illness.^16^,^17^ In the most recent population-based survey conducted in Qatar in 2019, a prevalence of tobacco smoking in adults was estimated at 21.5%.^18^ This rate is significantly lower than the rate reported in a previous study conducted between 1999 and 2000 (36.7%).^19^ However, these studies did not report the prevalence of smoking characteristics of people with mental health issues. Although it is well established that people with SMI are vulnerable to smoking, this vulnerability can also be influenced by sociocultural factors, which complicates comparisons of findings on smoking prevalence reported from other regions of the world.^20^

To date, no studies have examined the smoking patterns or the sociocultural factors that influence smoking among SMI people in Qatar. Filling this knowledge gap can help highlight the treatment needs and aid in advancing smoking cessation strategies for this vulnerable population. Thus, this study aimed to investigate the rates and patterns of smoking and tobacco cessation treatments in individuals with SMI attending outpatient psychiatric services in Qatar.

## Materials and Methods

This was a retrospective review of the electronic medical records (Cerner^TM^) of 346 outpatients (not hospitalized at a psychiatric facility within the previous year) attending a mental health clinic in Doha, Qatar. The sample consisted of a subset of individuals from another study^21^ which investigated the medical comorbidities of patients with SMI, including schizophrenia, major depressive disorder (MDD), bipolar disorder (BPD) and schizoaffective disorder (as per the Diagnostic and Statistical Manual of Mental Disorders).^22^ The subset of the sample of individuals with SMI for the present study consisted of those who had a documented smoking status in their medical records. Ethical approval for the main study was granted by the Institutional Review Board (IRB) of Qatar University (reference number: QU-IRB 501-E/15) and by Hamad Medical Corporation (HMC) Medical Research Center (MRC) (reference number: MRC 1526/2016). All procedures performed were in accordance with the ethical standards of both institutional IRBs and with the 1964 Helsinki declaration and its later amendments or comparable ethical standards. Patients’ consent to review their medical records was exempted by HMC MRC IRB as the research involved the collection of existing data and recorded in such a manner that subjects cannot be identified, directly or through identifiers linked to the subjects, such as name, address, phone number, health plan number, medical record number, social security number, or driver license number.

A comprehensive data extraction tool using an Excel^TM^ sheet was used to collect patient demographics, including gender, age, height, weight, and nationality. Laboratory investigations collected included systolic blood pressure (SBP), total cholesterol (TC), and high-density lipoprotein (HDL) cholesterol. Cerner^TM^ software’s open-text fields, including written assessments and progress notes, were hand searched to determine the individuals’ smoking status. Only patients with a present or past history of smoking were considered as holding a “smoking status”. If the open-text fields in the chart were left empty, the smoking status could not be confirmed, and thus these patients were excluded from the analysis. The extracted information from the open-text fields classifies patients’ smoking status as either “currently smoking” or “not currently smoking”, with the smoking of substances other than tobacco (eg, marijuana/cannabis and cocaine) expressly excluded. Additional data collected included psychiatric and non-psychiatric comorbidities, current pharmacotherapy, and tobacco cessation medications.

Descriptive analyses of demographic information and risk factors were performed. Categorical variables were expressed as frequencies and percentages, while continuous variables were expressed as means ± standard deviation (SD). Chi-square test for categorical variables and *t*-test for continuous variables were used to compare the demographic and smoking status of the cohort. Univariate analyses were conducted using Fisher’s exact test to determine the characteristic risks associated with smoking. Only covariates with p<0.05 were included in the multivariate logistic analyses to assess the independent predictors of smoking. Based on the univariate analysis, they were selected according to the procedure described by Hosmer and Lemeshow.^23^ Results were presented as odds ratios (OR) and 95% confidence intervals (95% CI). A false-positive type I or alpha error is set at 0.05. Data analysis was carried out through the SPSS^®^ statistical package (Version 23.0, SPSS Inc., Chicago, Illinois, United States).

## Results

**Figure 1:**
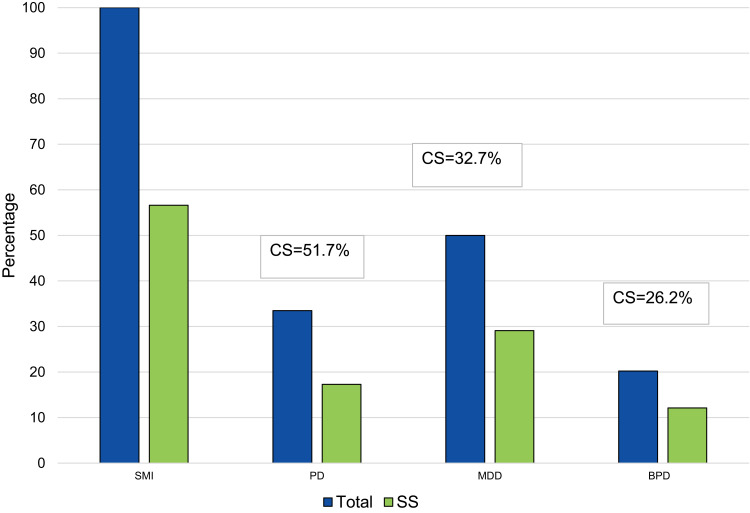
Smoking status in a sample of people with SMI (n=346) attending psychiatric services in Qatar.

**Abbreviations**: Total, Individuals in the study sample; CS, Current smokers; SS, Smoking status; SMI, Serious mental illness; PD, Psychotic disorders; MDD, Major depressive disorder; BPD, Bipolar disorder.

The medical charts of 346 outpatients were reviewed, of whom 196 (56.6%) had a documented smoking status, and thus included in this study. Table 1 presents the sociodemographic characteristics of the sample with a smoking status. Significantly more males than females were “current smokers” (62.9% versus 15.0%, respectively, p<0.001). As illustrated in Figure 1, among those with psychotic disorders (n=116), 60 (51.7%) had a documented smoking status, of whom 31 (51.7%) were current smokers; among those with MDD (n=173), 101 (58.4%) had a documented smoking status, of whom 33 (32.7%) were current smokers; and among those with BPD (n=70), 42 (60%) had a documented smoking status, of whom 11 (26.2%) were current smokers. The majority of the sample received antipsychotics or antidepressants (65.3% and 63.9%, respectively). Significantly more patients with psychotic disorders than those with any other SMI were “current smokers”, and this difference was statistically significant (p=0.006).

**Table 1:** Demographic Characteristics of the Study Sample

Characteristics	Smoking Status (n=196)	Currently Smoking (n=72)	Not Currently Smoking (n=124)	p-value^†^
Age, years, mean± SD	41.10 ± 11.90	40.54 ± 10.48	41.73 ± 12.65	0.534
Age groups, years, n (%)				
15–34	66	26 (39.4)	40 (60.6)	0.264
35–54	97	38 (39.2)	59 (60.8)	
55+	33	8 (24.2)	25 (75.8)	
Gender, n (%)				
Female	107	16 (15)	91 (85)	<0.001
Male	89	56 (62.9)	33 (37.1)	
Nationality, n (%)				
Qatari	81	26 (32.1)	55 (67.9)	0.259
Non-Qatari	115	46 (40)	69 (60)	
Psychiatric diagnosis, n (%)				
MDD	101	33 (32.7)	68 (67.3)	0.239
Psychotic disorder^a^	60	31 (51.7)	29 (48.3)	0.006
BPD	42	11 (26.2)	31 (73.8)	0.110
Psychotic disorder and MDD	4	3 (75)	1 (25)	0.141
Psychotic disorder and BPD	2	0 (0)	2 (100)	0.533
Medical comorbidities, n (%)				
Diabetes mellitus	38	11 (28.9)	27 (71.1)	0.349
Hypertension	24	4 (16.7)	20 (83.3)	0.040
Thyroid abnormality	15	2 (13.3)	13 (86.7)	0.090
Hyperlipidemia	28	8 (28.6)	20 (71.4)	0.404
Coronary artery disease	6	2 (33.3)	4 (66.7)	1
Other medical comorbidities	38	10 (26.3)	28 (73.7)	0.189
No. of comorbidities				
0	119	51 (42.9)	68 (57.1)	0.051
1	44	13 (29.5)	31 (70.5)	
≥ 2	32	7 (21.9)	25 (78.1)	
Psychiatric medications, n (%)				
Antidepressants	128	47 (36.7)	81 (63.3)	1
Antipsychotics	119	49 (41.2)	70 (58.8)	0.130
Mood stabilizers	31	9 (29)	22 (71)	0.418
Other psychiatric agents	40	13 (32.5)	27 (67.5)	0.585
Non-psychiatric medications, n (%)				
Hypoglycemics	33	8 (24.2)	25 (75.8)	0.117
Anti-hypertensives	29	6 (20.7)	23 (79.3)	0.062
Anti-lipidemic	28	7 (25)	21 (75)	0.207
Thyroid agents	10	1 (10)	9 (90)	0.097
Other non-psychiatric agents	40	17 (42.5)	23 (57.5)	1
Clinical/paraclinical factors				
BMI^b^, kg/m^2^, mean ± SD	32.10 ± 6.44	29.52 ± 6.44	31.88 ± 6.88	0.019
SBP^c^, mmHg, mean ± SD	127.51 ± 18.64	130.20 ± 14.51	125.96 ± 20.63	0.157
HDL^d^, mmol/L, mean ± SD	1.20 ± 0.35	1.11 ± 0.31	1.27 ± 0.36	0.001
Total cholesterol^d^, mmol/L, mean ± SD	4.98 ± 1.10	5.26 ± 1.22	4.81 ± 1.00	0.006

**Notes**: ^†^P-value calculated using Pearson’s Chi-square test or Student’s *t*-test. ^a^Including schizophrenia and schizoaffective disorder. ^b^Missing weight data for 16 patients. ^c^Missing SBP data for 2 patients. ^d^Missing HDL/Total cholesterol data for 32 patients.

**Abbreviations**: MDD, major depressive disorder; BPD, Bipolar disorder; BMI, body mass index; SBP, systolic blood pressure; HDL, high density lipoprotein cholesterol; SD, standard deviation.

Hypoglycemics and antihypertensives were the most common non-psychiatric medications used (15.0% and 14.5% of the sample, respectively). For those with documented smoking status for whom weight data were available (n=180), the mean ± SD BMI was 32.10 ± 6.44 kg/m^2^. Current smokers’ BMI was lower than the BMI reported in non-smokers (29.52 ± 6.44 versus 31.88 ± 6.88 kg/m2, respectively), and the difference was statistically significant (p=0.019). Only 12 (16.7%) of current smokers were receiving smoking cessation treatment.

**Figure 2:**
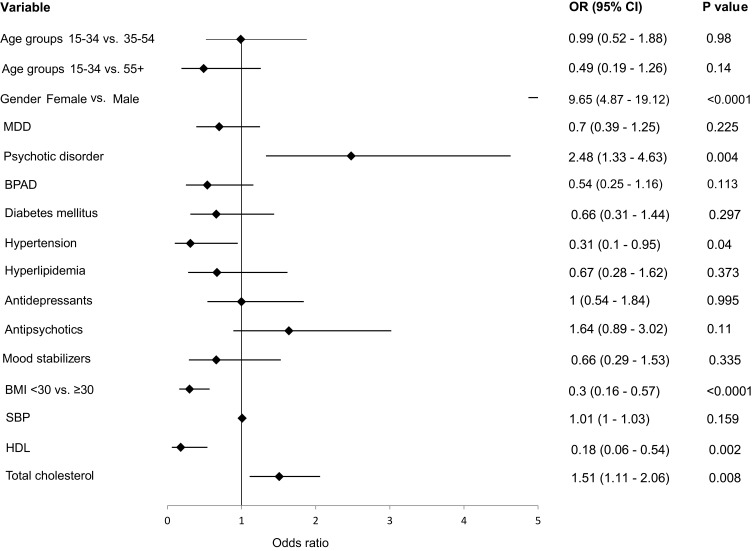
Forest plot for univariate analysis of the factors associated with current smoking in the SMI sample.

**Abbreviations**: OR, Odds ratio; MDD, Major depressive disorder; BMI, Body mass index; SBP, Systolic blood pressure; HDL, High density lipoprotein cholesterol.

Figure 2 illustrates the results of the unadjusted logistic regression analysis of factors associated with current smoking status. Positive and significant associations with current smoking were found for male gender [odds ratio (OR)=9.65; 95% CI=4.87–19.12; p<0.001], psychotic disorders (OR=2.48; 95% CI=1.33–4.63; p<0.004), and high levels (≥ 6.2 mmol/L) of total cholesterol (OR=3.30; 95% CI=1.34–8.09, p=0.008). Antipsychotic use was linked to a 64% greater smoking rate, although it did not achieve statistical significance (p=0.11). Negative and significant associations with current smoking were found for those with BMI <30 versus ≥30 kg/m^2^ [OR=0.30; 95% CI=0.16–0.57; p<0.001] and HDL-cholesterol [OR=0.18; 95% CI=0.06–0.54; p<0.008]. The adjusted logistic regression (AOR) analysis continued to show male gender (AOR=6.60; 95% CI=2.54–17; p<0.001) to be positively associated with smoking.

## Discussion

To our best knowledge, this study represents an initial attempt to gain insight into the prevalence of cigarette smoking and the associated factors that affect smoking behavior among people with SMI in Qatar. Overall, in the whole sample of SMI individuals attending an outpatient mental health clinic, the majority (56.6%) had a documented smoking status. This finding suggests that the smoking rate among SMI individuals in Qatar may be between 2–3 times higher than the rates reported for the general population (21.5%),^18^ and is in line with the majority of studies both, worldwide and those within the Middle East region, comparing the smoking rates between SMI samples and those in the general population.^7^,^16^,^17^,^24–29^

We also found that smoking was strongly associated with the male gender, which is broadly consistent with the findings in smoking prevalence studies, both in the general population and in people with SMI.^8^,^16^,^25^,^26^,^30–32^ Similarly, as with other studies in the Middle Eastern region,^27–29^ we found more significant differences in smoking rates in males versus females. This significant gender difference in smoking rates found in our study reflects the smoking patterns in the general population in Qatar, which showed a higher smoking prevalence of 36.6% in males versus 9.2% in females.^18^ These differences have been primarily attributed to sociocultural factors, as smoking is socially unacceptable among women in the Arab world in general, and Muslims in particular.^32^,^33^ There may also be under-reporting of smoking among women, fearing that their perception and image in the community could be affected.^27^,^34^,^35^ In addition, because social activities are largely gender-segregated, women may be less exposed to smoking communities than males.^27^,^33^,^36^ Thus, the lower current smoking prevalence reported by women with SMI, is most likely due to social and cultural factors, and possibly influenced by underreporting due to the fear of women of not being accepted in the society.

In our sample, smoking varied considerably across different psychiatric diagnoses. These differences were broadly consistent with prevalence rates derived from other studies worldwide, which reported the largest smoking rates among those with psychotic disorders.^8^,^24^,^26–29^,^37^ This diagnostic group in our sample also showed the highest rate of current smokers (51.7%) compared to those diagnosed with mood disorders (32.7% for MDD and 26.2% for bipolar disorder). The most widely accepted hypothesis for the higher prevalence of current smoking among people with psychotic disorders is self-medication; that is, because nicotine stimulates dopamine release in the nucleus accumbens, activating reward pathways, smoking may ameliorate negative symptoms in schizophrenia, and possibly also overcome dopamine blockade by antipsychotic medications.^38^,^39^ However, the self-medication hypothesis has been challenged in several studies, which suggest that the increased nicotine dependence observed in people with schizophrenia may represent an increased involuntary vulnerability that has little to do with any beneficial effects of nicotine on the cognitive and behavioral symptoms of schizophrenia.^40–42^ Furthermore, it has been suggested that this hypothesis may be negatively influencing the slower pace in the implementation of smoking cessation strategies and smoke-free policies in mental health facilities.^43^,^44^ It has also been reported that mental health providers have been permissive toward tobacco smoking among patients with SMI, particularly in the Middle East.^45^,^46^ The low percentage of current smokers in our sample of people with SMI receiving smoking cessation treatment (16.6%) may reflect the ambivalence that has been reported among mental health service providers in Qatar for implementing smoking cessation strategies.^46^ Studies are needed to investigate further the nature of this ambivalence, to help tailor smoking cessation strategies and smoke-free policies in Qatar’s mental health services.

The results also highlight essential clinical aspects of smoking that may affect people with SMI and are somehow interrelated. First, although the mean BMI was 31.05 kg/m^2^, the BMI of current smokers was statistically significantly lower, a finding that is consistent with the idea that cigarette smoking helps control body weight, although the mechanisms through which smoking decreases body weight are complex and incompletely understood.^14^ Second, current smokers were three times more likely to have high levels of total cholesterol (≥ 6.21 mmol/L) and less likely to have higher HDL-cholesterol levels (>0.75 mmol/L for males; >0.91 mmol/L for females). These findings also support results from clinical trials that suggest that cigarette smoking is associated with a adverse lipid profiles, characterized by more elevated total cholesterol and lower HDL cholesterol levels.^47^ Third, antipsychotic use was linked to a 64% greater current smoking rate, which highlights not only that the largest smoking rates are found among those with psychotic disorders, but also that there is a relationship with important cardiometabolic risk factors, such as BMI and cholesterol, that will need to be further explored and considered when establishing smoking cessation interventions, particularly in patients with psychotic disorders using antipsychotic medication.^48^

The study’s limitations correspond to the small sample size due to its retrospective nature and an overall insufficient documentation of tobacco use pattern in the medical records, such as shisha smoking, overall tobacco consumption, and smoking cessation attempts or treatment history. This may certainly limit the generalizability of the study findings. However, considering that in Qatar, individuals with a diagnosis of SMI receive ongoing care primarily from outpatient clinics located within the only psychiatric facility in the country, the sample may have some degree of representativeness. Finally, the archival data did not include information concerning the patient’s socioeconomic status and education, which may also influence high smoking rates among psychiatric patients. Thus, it is recommended to replicate the study on a larger scale and using a matched population without SMI to draw more generalizable conclusions. Future studies would also benefit from collecting data prospectively. Despite these limitations, the results from this study add to the scant evidence documenting smoking prevalence among people with diagnosed SMI in Qatar.

## Conclusion

More than half of a sample of people with SMI attending psychiatric services in Qatar had documented smoking status. Still, only a few current smokers were on smoking cessation treatment. Our findings suggest that smoking is a significant health concern, particularly for individuals with psychotic disorders, who presented with the highest current smoking rates. Due to this population’s higher morbidity and mortality, providers caring for these vulnerable subjects can help by ensuring interventions are prioritized and offered, documented, and regularly followed up.
